# Minimally invasive treatment of early, good prognosis breast cancer—is this feasible?

**DOI:** 10.1093/bjr/tqae028

**Published:** 2024-02-03

**Authors:** Mhairi Mactier, Stuart A McIntosh, Nisha Sharma

**Affiliations:** Golden Jubilee National Hospital, Clydebank G81 4DY, United Kingdom; Patrick G Johnston Centre for Cancer Research, Queen’s University Belfast, Belfast BT9 7AE, United Kingdom; Breast Unit, St James Hospital, Leeds LS9 7TF, United Kingdom

**Keywords:** breast cancer, breast screening, minimally invasive surgery, vacuum-assisted excision, cryotherapy, radiofrequency ablation

## Abstract

Breast cancer screening programmes frequently detect early, good prognosis breast cancers with significant treatment burden for patients, and associated health-cost implications. Emerging evidence suggests a role for minimally invasive techniques in the management of these patients enabling many women to avoid surgical intervention. Minimally invasive techniques include vacuum-assisted excision, cryoablation, and radiofrequency ablation. We review published evidence in relation to the risks and benefits of each technique and discuss ongoing trials. Data to date are promising, and we predict a trend towards minimally invasive treatment for early, good-prognosis breast cancer as technical skills, suitability criteria, and follow-up protocols are established.

## Background

In the United Kingdom, women aged 50 to <71 years are invited for screening mammography every three years. The risks and benefits of breast screening have long been controversial, resulting in the publication of the UK Independent Panel Report on Breast Cancer Screening in 2012.^1^ This review examined published literature and concluded that for every three cases of breast cancer diagnosed and treated, one breast cancer death was prevented. Although the incidence of breast cancer was increasing prior to screening, the introduction of mammographic screening programmes resulted in a marked increase in the incidence of smaller (<2 cm) tumours, with a smaller decrease seen in the incidence of larger (≥2 cm) tumours.^2^ Further analysis of mortality trends suggests that women with smaller tumours are more likely to be over-diagnosed rather than having earlier detection of tumours which are destined to become large, with “overdiagnosis” defined as a diagnosis of a tumour which never becomes clinically apparent during a patient’s lifetime. Overdiagnosis may be due to either non-progressive disease, or due to indolent cancers which progress so slowly that competing causes of death negate their clinical significance, with estimates of the contribution of each cause varying with age. The consequence of the “overdiagnosis” of such small tumours is a treatment burden resulting from surgical and adjuvant treatments which may have minimal survival benefit. However, it is not currently possible to reliably identify individual tumours as overdiagnosed.

The reduction in breast cancer mortality seen in the decades since the initiation of screening may be attributable to improvements in systemic therapy rather than being entirely due to earlier detection of disease.^3^ Smaller tumours showing biologically favourable characteristics (oestrogen/progesterone receptor positive, HER2 negative tumours) are associated with almost 100% survival at 10 years.^4^ Our understanding of tumour biology and associated prognosis have been further improved by the introduction of molecular assays. For example, the Oncotype Dx 21 gene assay was developed in 2004 and is used to calculate the risk of distant recurrence at 10 years.^5^ The test assigns a recurrence score between 0 and 100, and patients are categorized into three groups—low (<18), intermediate (18-30), and high risk (≥31). The TAILORx trial demonstrated patients with low recurrence scores had a similarly excellent prognosis irrespective of tumour size.^6^ It is clear therefore that the tools exist to identify those small tumours with favourable biology and a good prognosis, many of which are likely to be screen detected.

It is therefore necessary to tailor treatment, with its attendant morbidities, appropriately to the individual tumour. There are published data to suggest that patients who are diagnosed in a screening programme receive less intense treatment than those patients never screened (or not recently), even after correcting for overdiagnosis.^7^ Given the potential advantages of this in reducing the treatment burden for the individual as well as healthcare provider costs, there is an urgent need to consider optimizing treatment and minimizing the burden for patients with small, good-prognosis screen-detected tumours. This includes the consideration of alternative, minimally invasive treatment pathways for such cancers.

## Surgical treatment for screen detected breast cancers

Current clinical guidelines advise the same treatment pathways for screen detected and symptomatic cancers. Patients undergo surgical excision (with or without localization techniques) and axillary staging. Most screen detected breast cancers are managed with breast conserving surgery. Associated morbidity is low but not insignificant—complications include surgical site infection (1.4%), haematoma (2.6%), seroma (11.5%),^8^ and up to 28.3% of patients report poor cosmetic outcomes with associated psychosocial morbidity following breast conserving surgery.^9^ Furthermore, following histopathological assessment of resection margins, up to 25% of women return to theatre for a repeat procedure to achieve clear margins. Such re-operations add to associated morbidity as well as resulting in increased healthcare costs.^10^ Close or involved margins (≤2 mm) are associated with local and/or distal recurrence,^11^ however, 30%-65% of women demonstrate no residual disease on re-excision,^12^ and this does not impact survival outcomes.^13^ Axillary staging has additional risks of shoulder stiffness (17.1%-29.8%), lymphoedema (5.9%-23.6%), and chronic pain (21.7%-32.9%).^14^ The ACOSOG Z0011 trial^15^ found low-burden axillary disease can be treated by systemic therapy, avoiding completion axillary dissection without adversely impacting breast cancer mortality, and recent results from the SOUND trial have shown non-inferiority of no axillary surgery in a clinically node-negative axilla compared to sentinel lymph node biopsy with 98% disease-free survival at 5 years.^16^

## Minimally invasive treatment options

With increased detection of early, good prognosis breast cancers, we need alternative treatment modalities to offer patients which reduce both treatment intensity and toxicity whilst maintaining optimal disease control. There is no prospective evidence that open surgical intervention of these tumours is required. Numerous minimally invasive techniques have been described—in this review, we are going to focus on vacuum-assisted excision (VAE), cryoablation, and radiofrequency ablation (RFA).

### Vacuum-assisted excision

**Figure 1.**
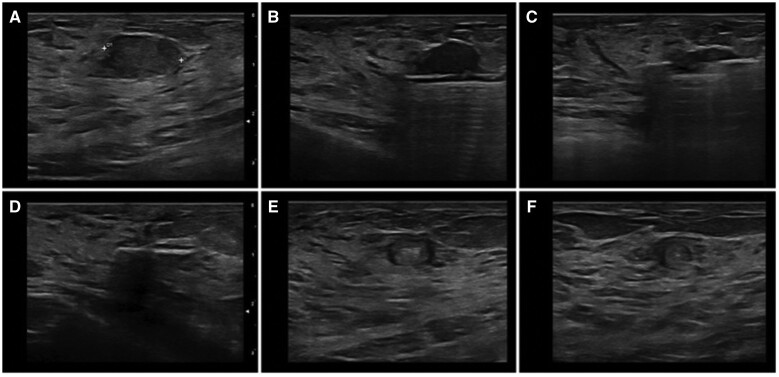
Step-by-step ultrasound-guided vacuum-assisted excision of a fibroadenoma. (A) solid hypoechoic lesion, with regular shape and a circumscribed margin (B, C) US guided 8G vacuum-assisted excised (D) lesion has been completed removed and metallic marker clip release (E) axial follow-up scan at 12 months (F) longitudinal follow-up scan at 12 months.^18^

Vacuum-assisted excision is a non-surgical technique used worldwide for both diagnosis and management of benign breast lesions and lesions of uncertain malignant potential. However, VAE is not currently in use for the treatment of breast cancer without clinical trials. Initially described for diagnosis only, vacuum-assisted biopsy (VAB) has evolved into VAE. A larger calibre needle (up to 7G) allows multiple cores to be taken with the needle remaining *in situ* after a single pass. This obtains an increased volume of tissue enabling histopathological assessment of an entire mass and provides additional information which cannot be achieved via fine-needle aspiration or core-needle biopsy. Using stereotactic or ultrasound guidance, the procedure is carried out under local anaesthetic in the outpatient setting (Figure 1). The technique is well accepted by patients—in a survey of 189 females, 90% of patients preferred VAB to surgical biopsy, noting that it took less time and provided ‘better cosmetic results’.^17^ It should be noted however that VAB may involve less extensive tissue sampling and a smaller bore needle than VAE (patients in this series had biopsies with 9G and 11G needles, whereas VAE might utilize 7G-11G needles). Consequently, VAE may be less well-tolerated than VAB, although there are no published data to support this hypothesis.

The use of VAE in the excision of benign breast lesions has been supported by the National Institute for Health and Care Excellence (NICE) since 2006.^19^ A meta-analysis of 26 studies investigating the efficacy and safety of VAE in benign lesions found complete resection rates following VAE of 93% (95% CI, 0.90-0.95) and recurrence rates of 39% (95% CI, 0.02-0.09). Complication rates following VAE of post-procedure haematoma, pain, and ecchymosis were reported as being 92% (95% CI, 0.07-0.13), 82% (95% CI, 0.05-0.13) and 75% (95% CI, 0.05-0.12) respectively, significantly higher than those for open surgical approaches.^20^ Lower rates of haematoma were reported in a further meta-analysis which suggested rates of around 2.6%-3.3% following open surgery. However, it is unclear whether the nature and severity of the complications reported in the series of the two techniques are comparable—as surgical series often only report complications requiring operative evacuation whereas the majority of haematomas following VAE are treated conservatively. For future studies evaluating VAE in comparison with surgery, it will be important to incorporate robust comparisons of complications between techniques, coupled with patient reported outcomes to assess acceptability and tolerability.

Building on this there are now substantial published data to support the safe use of VAE in the management of lesions of uncertain malignant potential (“B3 lesions”) with complete excision rates reported of up to 93.6%,^18^ post-VAE imaging confirming complete lesion removal in >90% cases.^21^ VAE is now included in the UK NHS Breast Screening Clinical Guidelines for management of B3 lesions.^22^

Vacuum-assisted excision is not currently in use for the treatment of either invasive or *in situ* breast cancer, although this is an area of interest and there are data to suggest that VAE may be able to fully excise small tumours.^23^ The ongoing UK SMALL trial is a prospective, randomized (2:1) phase III trial assessing VAE versus open surgery in patients with small, biologically favourable screen detected breast cancers.^24^^,^^25^ In the VAE arm, a clip is placed at the tumour bed, and completeness of excision is assessed radiologically. Any patients found to have a residual radiological disease or grade three pathology will be offered open surgical excision. All patients will have adjuvant radiotherapy to the breast and endocrine therapy as per local protocols with mammographic follow-up for 5 years. Co-primary outcomes of this study are non-inferiority rates of VAE in avoiding a second procedure to achieve complete resection (≤10% accepted), and local recurrence rates at 5 years. Secondary outcomes include psychological and aesthetic impact of VAE, and a full health economic analysis will also be undertaken (Table 1).

**Table 1.** A summary of the main completed and ongoing clinical trials for these three minimally invasive techniques.

Study	Minimally invasive technique	Study type	Inclusion criteria	Status
SMALL^24^^,^^25^	VAE	Phase III randomized-controlled trial	Females >47 years Screen-detected breast cancer ≤15 mm maximum tumour diameter No mammographic microcalcification Unifocal disease Grade 1 ER strongly positive (Allred ≥7) PR strongly positive (Allred ≥7) HER2 negative (0 or 1+ on IHC, or 2+ and negative on ISH) Normal axillary ultrasound/equivocal axillary ultrasound with benign FNAC or core biopsy No previous ipsilateral breast cancer or DCIS	Recruiting *(383 patients as of January 2024)*
ACOSOG Z1072 (Alliance)^30^	Cryoablation	Phase II single-arm trial	Unifocal invasive ductal carcinoma ≤2 cm <25% intraductal component Tumour enhancement on MRI	Results published 2016 Successful ablation 92% patients Residual *in situ* disease in 24.1% Recommendation: Improved imaging and better patient selection required
ICE3^31^^,^^33^	Cryoablation	Phase II single-arm trial	Females ≥50 years Unifocal primary breast cancer ≤1.5 cm Invasive ductal breast carcinoma Nottingham grade 1-2 (nuclear/mitotic score <2) ER+, PR+, HER2- Lesion visible on ultrasound at time of treatment No previous ipsi/contralateral breast carcinoma	Interim 3-year analysis published 2022 Ipsilateral breast tumour recurrence rate 2.06% at 34.86 month follow-up >95% patients, and 98% clinicians satisfied with cosmetic result Recommendation: Further study required, eg, randomized-controlled trial
FROST^32^	Cryoablation	Phase II single-arm trial	Age ≥50 years Unifocal primary breast carcinoma ≤1.5 cm T1N0 Hormone receptor+, HER2- Lesion visible on ultrasound Patient agrees to 5 years minimum adjuvant endocrine treatment	Follow up phase; results awaited
RAFAELO^39^	Radiofrequency ablation (RFA)	Phase III single-arm trial	Single focus invasive breast cancer <1.5 cm N0M0 No previous treatment for breast cancer	Follow up phase; results awaited

### Cryoablation

**Figure 2.**
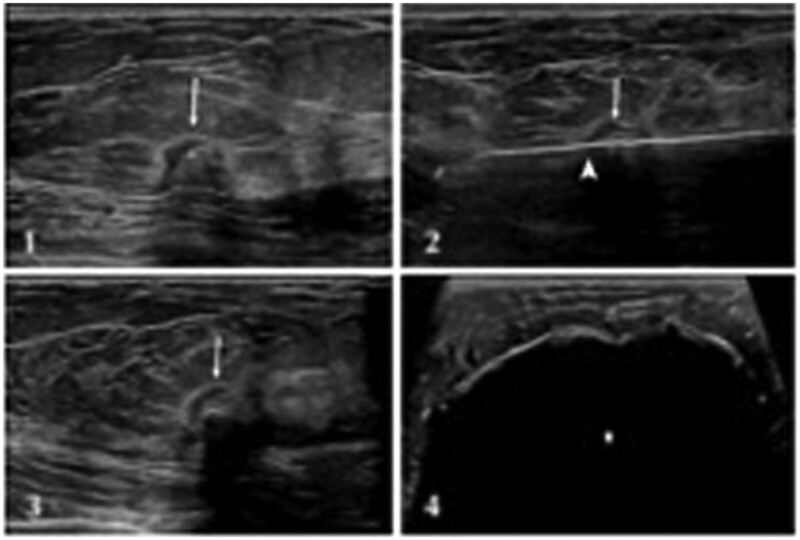
(1) 1.1 cm grade II ER/HR+ HER2-intraductal carcinoma (2 and 3) long-axis and short-axis view of cryoprobe placement within the tumour [arrowhead denotes edge of tumour; caliper (+) denotes cryoprobe tip] (4) long-axis view of ice ball (*) enveloping the tumour. Adapted from Regen-Tuero et al.^29^

Cryoablation uses the cytotoxic effects of cold to create tumour necrosis. The ablation process has three phases: first freeze; passive thawing; and second freeze. Freezing tissues at lethal temperatures causes increased intracellular osmolarity and cellular dehydration, whilst passive thawing causes cellular swelling and subsequent rupture. Indirect cryoablation also results in microthrombus formation and ischaemia. The second freeze is necessary to enhance the damaging effects of cold and expand the area of tumour necrosis—this is indicated for malignant lesions to create an “ice ball” extending at least 1 cm beyond the tumour margins. The procedure takes less than 45 min to complete, can be performed under ultrasound, CT, or MRI guidance (Figure 2), and typically requires less local anaesthetic compared to other minimally invasive techniques due to the synergistic cooling effect of the probe. Adverse side effects include low capability to limit ablation areas due to concerns of possible fat necrosis and/or infection as well as minor effects including ecchymosis, skin burns, and minor breast pain.^26^ Skin rash at the time of intervention is common, but this typically resolves within 8 h.^27^ In a systematic review of 161 patients, there were only three cases of seroma (1.9%), one case of skin retraction (0.6%), and one case of skin necrosis (0.6%), with satisfactory cosmesis reported in 99% cases.^28^ Following cryoablation, the damaged cancer cells remain *in situ* in the treated area—this is less likely to be accepted by patients with symptomatic disease and can impose challenges for surveillance imaging. Early detection of local recurrence is of paramount importance for disease-free survival and to date, there is no consensus on the best method of radiological follow-up for patients following cryoablation therapy.^26^

The use of cryotherapy in benign breast lesions and metastatic deposits is well described however substantive evidence supporting the use of cryoablation for invasive breast cancer is lacking. The most important trial to date exploring the use of cryoablation in the treatment of early-stage breast cancer was the ACOSOG Z1072 trial^30^ (Table 1). Patients with unifocal invasive ductal carcinoma ≤2 cm underwent cryoablation, followed by surgical resection of the primary tumour within 28 days. Of 87 cancers treated, ablation of the target lesion was successful in 92% of patients however residual invasive and/or *in situ* disease was identified in 24.1%. This was attributed to multifocal disease outside of the targeted cryoablation zone. Typically, this technique would not be advised in patients with ductal carcinoma *in situ* (DCIS) or lobular cancer due to its multifocal nature.

Two ongoing trials in the United States are exploring this further: ICE3 (Cryoablation of Low Risk Small Breast Cancer)^31^ and FROST (Freezing Instead of Removal Of Small Tumours).^32^ Patients with unifocal primary invasive disease ≤1.5 cm undergo cryoablation treatment, with a follow-up ultrasound core biopsy at 6 months to confirm the absence of residual viable disease. Patients then receive a minimum of 5 years of endocrine treatment and serial breast imaging. Adjuvant radiotherapy is mandatory for patients aged 50-69 but optional for those ≥70 years in FROST, and as per local policies in ICE3. Any subjects found to have residual or recurrent disease will be offered standard surgical excision. The primary endpoint for both studies is 5 years local recurrence rate. ICE3 has released a three-year interim analysis with promising results: ipsilateral breast cancer recurrence following cryoablation was 2.06% and more than 95% of patients and 98% of clinicians reported satisfaction with cosmetic results.^33^ The optimal method of radiological follow-up is not clear; however, the FROST trial is carrying out mammography, ultrasound, and MRI for all patients which may advise guidelines on the best method of radiological follow-up post-cryoablation. FROST is due to report results later this year. Of note, both these trials are single-arm phase II studies and there are currently no randomized controlled trials exploring the use of cryotherapy in early breast cancer.

### Radiofrequency ablation

**Figure 3.**
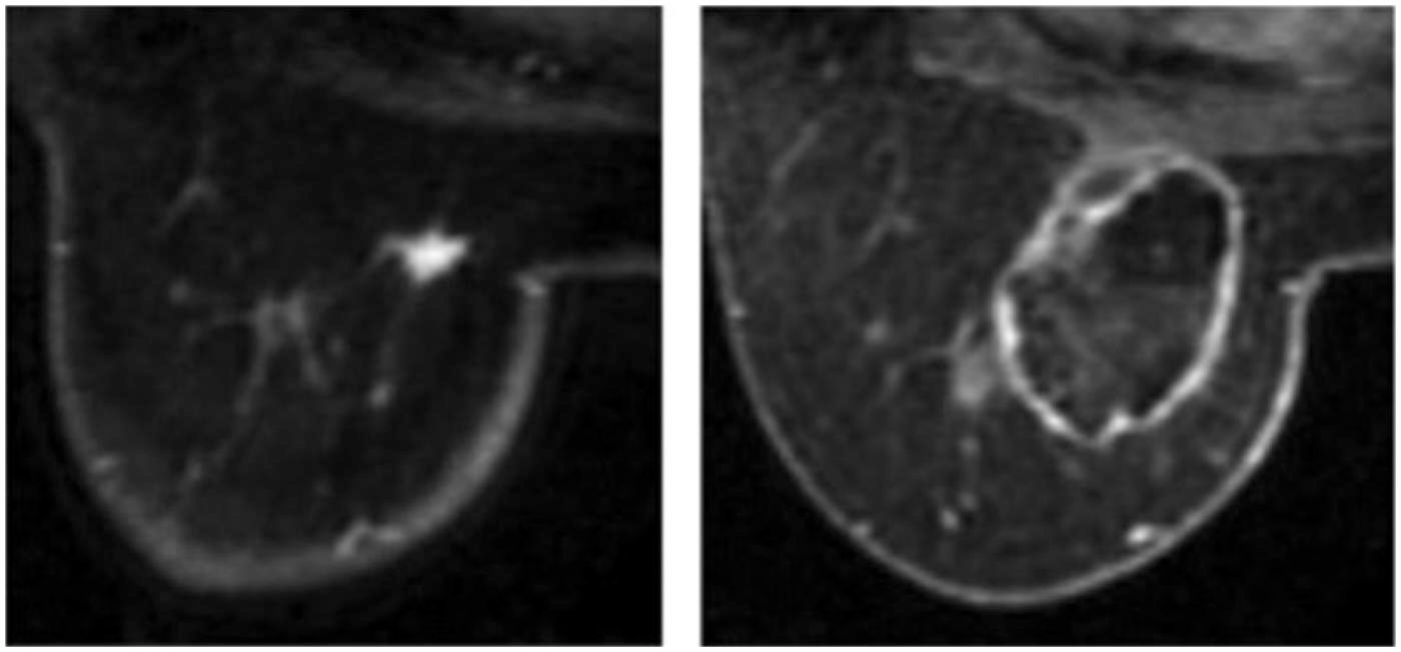
(Left) MRI before RFA; (Right) MRI after RFA.^34^

Radiofrequency ablation uses low-frequency radio-waves with long wavelengths to generate heat around a needle electrode causing localized coagulative tissue necrosis. Cancer cells contain more water than normal cells with fragile neoplastic vasculature—their susceptibility to heat-induced thrombosis results in localized necrosis of malignant tissue with minimal destruction of surrounding healthy cells. RFA can be performed under local anaesthetic with ultrasound, CT, or MRI guidance—ablation is observed with the formation of echogenic microbubbles (Figure 3). Reported side effects include skin burns (<3% incidence), inflammation of the breast (2.5%), nipple retraction (0.5%), and pneumothorax (0.5%).^34^ Like cryotherapy, the treatment goal is to ablate the whole lesion plus a 1 cm tissue rim, which reduces its suitability for lesions close to the skin, nipple, chest wall, or implant. Heat is not supplied by the probe itself therefore a limited volume of tissue can be ablated at one time and/or multiple probes are required for larger lesions.

Radiofrequency ablation is used for both treatment and palliation in numerous solid tumours including hepatocellular carcinoma, non-small cell lung cancer, and renal neoplasms.^35^ Technical success of RFA in the treatment of breast cancer was first described in 1999. Evidence suggests RFA is best used for smaller lesions ≤2 cm. Retrospective analysis of 386 patients across 10 institutes undergoing RFA found higher local recurrence rates in patients with initial tumour size >2 cm compared to those with ≤2 cm (10% versus 2.3%). Disease-free survival at 5 years following RFA was 97%, 94%, and 87% in patients with initial tumour sizes <1 cm, 1.1-2 cm, and >2 cm, respectively.^36^ These results are supported by a meta-analysis of 401 lesions ≤2 cm,^34^ however, there is an ongoing requirement for prospective studies to validate these findings and help guide clinical practice.

In a single-arm prospective study, Kinoshita et al treated 58 localized breast cancers ≤1 cm with RFA alone and followed them up with clinical examination, diagnostic imaging (ultrasound, MRI, and mammography), and VAB at 3, 6, and 12 months. All patients with suspected residual disease or incomplete ablation were offered surgical resection. Ninety percent of patients had a complete ablation rate, and there were no episodes of ipsilateral breast cancer or distal recurrence at a median follow-up of 1832 days.^37^ Garcia-Tejedor et al randomized 40 patients to RFA and lumpectomy, or lumpectomy alone, reporting positive margins in 55% of the control group, but only 20% of the RFA group. However, local breast inflammation and infection after surgery were higher in the RFA group (40% versus 5%, and 15% versus 0 respectively). No cases of ipsilateral recurrence were identified at a median follow-up of 25 months.^38^ These results led to the phase III, multicentre RAFAELO trial (Radiofrequency Ablation Therapy for Early Breast Cancer as Local Therapy).^39^ This is a single-arm study assessing the safety and efficacy of RFA in early-stage breast cancer; results will be compared with a previous randomized controlled study of breast conserving surgery. Women with localized breast cancer ≤1.5 cm undergo RFA and sentinel lymph node biopsy under general anaesthetic. Following adjuvant whole-breast radiotherapy, patients will have follow-up imaging and VAB at three months. If there is evidence of histological residual lesion, patients will undergo open surgical resection. The primary endpoint is 5-year ipsilateral disease-free survival, and secondary endpoints include overall survival, distal disease-free survival, adverse events of RFA, and tumour viability after RFA. A total of 372 patients were recruited to this trial making it the largest prospective trial assessing the use of RFA in early breast cancer to date. Results should be available later this year, but analysis of results should consider concurrent use of general anaesthesia and sentinel lymph node biopsy. To our knowledge, there are no large, prospective randomized-controlled trials exploring the use of RFA in early breast cancer.

## Comparison of techniques

A meta-analysis of 1168 breast lesions compared technical success, technique efficacy, and complications associated with minimally invasive image-guided percutaneous ablation methods. RFA is the most used method (50%), with cryoablation used in only 13%. Pooled technical success was 96% (96% in radiofrequency and 95% in cryoablation), whilst pooled technique efficacy was suboptimal at 75% (67%-81%). Technique efficacy was significantly better in patients who underwent RFA and cryoablation compared to laser, microwaves, and high-intensity focused ultrasound, and complication rates were low across the techniques (6%-8%). Patient-reported outcomes were not considered in this review.^40^ Whilst this remains the largest published meta-analysis comparing different minimally invasive techniques, there are some limitations. The mean sample size was small (*n* = 24), the type of staining method used to assess the completeness of ablation at histology was not accounted for and in terms of reporting complication rates—there was an inhomogeneous description between the studies, thus patients were largely grouped into major and minor categories.

Manenti et al compared RFA (*n* = 40) and cryoablation (*n* = 40) in the treatment of early breast cancer. All patients subsequently underwent open surgery 30-45 days following ablation treatment and no episodes of local recurrence were identified at 18-month follow-up. Cryotherapy was considered the preferred method due to the analgesic effect of freezing with associated with better patient compliance.^27^ More recently, van de Voort et al have opened the THERMAC trial comparing the efficacy of RFA, microwaves, and cryoablation for early-stage breast cancer with the goal of selecting a technique for a subsequent phase III comparative study.^41^ There is currently no evidence, nor any ongoing clinical trials comparing percutaneous ablation techniques and VAE.

## Discussion

When comparing minimally invasive techniques, it is important to acknowledge that whilst VAE will excise the tumour, both percutaneous ablation techniques leave a mass *in situ*. This may be difficult for patients, particularly those with palpable lesions, who will continue to complain of a lump, and pose challenges for both clinical and radiological follow-up. In addition, minimally invasive techniques do not allow for formal histopathological assessment of surgical margins. These considerations, together with concerns about the tolerability of minimally invasive approaches may mean that some patients would prefer one treatment approach over the other. It is important that future trials evaluating minimally invasive approaches explore patient preferences, as is taking place within the Qualitative Recruitment Intervention in the SMALL trial.^24^^,^^25^

Whilst close or involved margins are associated with local recurrence, we also know that a clear margin does not mean there is no residual disease in the breast, nor does this impact survival outcomes. Holland et al examined a series of patients undergoing mastectomy for clinically and/or radiologically unifocal disease.^42^ This series included a cohort of patients (18% of the total) with screen-detected disease. In 130 patients with T1 tumours, 17% of cases had tumour foci within 2 cm of the index lesion, with a further 42% having additional foci >2 cm from the reference tumour. There was no statistically significant difference in the incidence of multifocal tumours with reference to lesions </> 2 cm.

Improved imaging techniques and the use of contrast imaging modalities (such as magnetic resonance imaging and contrast-enhanced spectral mammography) may be valuable in identifying patients with such occult foci of disease.^43^^,^^44^ Patients with occult multifocal disease may not be suitable for such percutaneous approaches to treatment, and future studies of minimally invasive techniques may require assessing the role of contrast imaging for patient selection in this context.

However, in an era of effective systemic therapies, what is important is not necessarily the volume of tumour left behind but rather the clinical significance of such microscopic occult disease. In a large retrospective population-based cohort study of 31 199 patients, Vos et al report a local recurrence rate of 2.9% in patients with focally positive margins at breast conserving surgery compared to 2.3% in patients with negative margins (*P* = .099). In the context of radiotherapy, re-excision of positive margins was not associated with improved disease-free or overall survival when compared to omitting re-excision (87.1% versus 86%, adjusted HR 0.83 [95% CI, 0.59-1.17] and 92.1% versus 92.7%, adjusted HR 1.17 [95% CI, 0.87-1.59] respectively).^13^ Findings from the IMPORT LOW randomized controlled trial report very low local recurrence rates in low-risk breast cancer patients receiving adjuvant radiotherapy, and this study found non-inferiority survival outcomes for both reduced-dose and partial-breast radiotherapy compared to whole-breast radiotherapy at 5-year follow up (0.2%, 0.5%, and 1.1%, respectively, *P* = .003-.016).^45^ These findings support the hypothesis that minimally invasive treatment approaches in the context of standard multidisciplinary adjuvant therapies can achieve acceptable oncological outcomes.

However, there is considerable interest among researchers in studies examining de-escalation of such adjuvant therapies, particularly radiotherapy. Studies such as PRIMETIME,^46^^,^^47^ PRECISION,^47^ EXPERT,^48^ DEBRA,^49^ and IDEA^50^ are examining the omission of radiotherapy in biomarker-determined low-risk disease, and the PROSPECT trial used MRI to identify patients with true unifocal disease who could potentially avoid radiotherapy.^51^ Currently, such studies require patients to undergo standard surgery in order to de-escalate the radiotherapy component of their treatment. Such de-escalation may have benefits for patients in terms of reducing treatment burden, and to healthcare systems in terms of reducing resource use. The implementation of hypofractionated radiotherapy regimens, reducing treatment to 5 fractions, however, partly mitigates against these benefits. There are fewer studies evaluating the omission or de-escalation of endocrine therapy, although both LALEAST^52^ and LESS^53^ are evaluating shorter durations of adjuvant endocrine therapy in the setting of low-risk disease.

What is clear, however, is that patients may well have different preferences for treatment in line with their own beliefs, cultures, and circumstances. This is borne out by patient surveys which confirm that different patients wish to de-escalate different components of their therapy should it be safe to do so. A recent international patient survey demonstrated that 30% of patients would prefer to omit chemotherapy, 26% endocrine therapy, 17% surgery, and only 8% radiotherapy (unpublished data; Potter et al SABCS 2023). It is therefore important to generate data to support the de-escalation of each therapeutic modality to allow breast cancer patients to make informed choices regarding treatment.

## Conclusions

Minimally invasive techniques may offer a suitable alternative to surgical excision for low-risk, early breast cancers, particularly those small, good-prognosis tumours likely to be overtreated currently, with the attendant morbidities that ensure. These techniques are less invasive for patients, deliver good results, and are a cost-effective intervention for healthcare providers. VAE offers the benefit of excising the lesion as whole, whilst cryoablation and RFA destroy cancer cells with localized effects, although all may have a role to play in the management of the low-risk disease. Furthermore, such approaches need to be viewed in the context of the multidisciplinary treatment of breast cancer and take into account the preferences of patients to de-escalate different treatment modalities. High-quality evidence from prospective studies including randomized controlled trials will be required before adopting these approaches into routine clinical practice.
